# Stress, mental symptoms and well-being in students: a gender analysis

**DOI:** 10.3389/fpsyg.2024.1492324

**Published:** 2024-12-11

**Authors:** María-José del Pino, M. Pilar Matud

**Affiliations:** ^1^Department of Sociology, Universidad Pablo de Olavide, Seville, Spain; ^2^Department of Clinical Psychology, Psychobiology and Methodology, Universidad de La Laguna, San Cristóbal de La Laguna, Spain

**Keywords:** gender, stress, coping styles, mental symptoms, well-being, self-esteem, social support, students

## Abstract

**Introduction:**

Stress is a major problem among students, threatening their health and well-being. The aim of the research is to analyze the sources of stress in students and to investigate whether there are gender differences and differences between university and non-university students in stress, coping styles, mental symptoms and well-being. A second aim is to know the relevance of age, education, stress, coping styles, self-esteem and social support on mental symptoms, psychological well-being and life satisfaction of boys and girls.

**Methods:**

The study was cross-sectional. The sample consisted of 1,426 students between the ages of 16 and 26.

**Results and discussion:**

The results of the ANOVAs showed that although there were some gender differences, being a university student or not explained more variance than gender. University students had more chronic stress than non-university students. However, they were more satisfied with their studies, had healthier coping styles, fewer mental symptoms, and greater well-being. For both genders, the main predictor of more mental symptoms was a higher emotional coping style. This was followed by lower self-esteem, a higher number of stressful life events, and higher chronic stress. Higher study dissatisfaction was associated with lower psychological well-being and lower life satisfaction. Higher chronic stress was associated with lower life satisfaction. It is concluded that stress and coping styles are relevant to students’ mental health. The results of this study are relevant to the design of policies, strategies, and programs to improve students’ mental health and well-being.

## 1 Introduction

Mental disorders are among the top ten leading causes of disease burden worldwide (GBD 2019 Mental Disorders Collaborators, 2022). Mental disorders are common among students (Auerbach et al., 2018; Pascoe et al., 2020; Lipson et al., 2022; Mayya et al., 2022; Pérez et al., 2023), a population considered key to a country’s economic growth and success (Auerbach et al., 2018). The onset of many mental disorders occurs during adolescence (Kessler et al., 2007; Auerbach et al., 2018) or early adulthood (Altwaijri et al., 2020). Mental health problems during adolescence and young adulthood are associated with negative outcomes, including potentially lifelong health problems, lower academic achievement, and lower likelihood of employment (Fletcher, 2010; Mojtabai et al., 2015; Patten, 2017).

Among the most consistent patterns of differences in mental health problems are gender differences in the prevalence of some mental disorders. There is evidence that internalizing problems, such as anxiety and depression, are more common in women than in men, while externalizing problems, such as antisocial personality and substance use or dependence, are more common in men (Seedat et al., 2009; Rosenfield and Mouzon, 2013; Kuehner, 2017). Symptoms of depression and anxiety and suicidal ideation have increased in recent cohorts of adolescents and college students (Duffy et al., 2019; Twenge et al., 2019; Lipson et al., 2022; Parodi et al., 2022; Samek et al., 2024). Although students were a sector particularly affected by the COVID-19 pandemic (von Keyserlingk et al., 2022; Matud et al., 2023; Wang et al., 2024), increased rates of depression and anxiety had been reported prior to this pandemic. Furthermore, the increase was greater in girls than in boys (Duffy et al., 2019; Hinze et al., 2024; Twenge et al., 2019).

College students face many challenges and changes as it is a time of great instability with changes in romantic relationships, peer groups, course selection, and career choices (Auerbach et al., 2018). Adolescence is a time of transformation (Blakemore, 2019). It is an important period, both personally and socially, as it allows young people to develop skills and gain experiences that prepare them for healthy and productive adult lives (Coyne-Beasley and Halpern-Felsher, 2020). One of the stresses students face during these life stages is academic stress, “a phenomenon characterized by the overwhelming pressure and anxiety experienced due to the demands of academic life” (Dagli et al., 2024, p. 1). Academic stress is thought to be the result of interactions between environmental stressors and student’s appraisals and/or reaction to them (Reddy et al., 2018; Perrella et al., 2024). While academic stress is suggested to be intensified in college due to increased workload, there is also evidence that such stress is pervasive among high school students, affecting not only their academic lives, but also their health and well-being (Deb et al., 2015; Subramani and Venkatachalam, 2019; Pascoe et al., 2020; Barbayannis et al., 2022; Mayya et al., 2022). Academic stress can reduce motivation and academic achievement, and increase the risk of dropping out of school, with long-term consequences (Pascoe et al., 2020). Research on gender differences in academic stress has been inconclusive, with some studies finding that academic stress is higher for girls (Anniko et al., 2019; Barbayannis et al., 2022) and others for boys (Mayya et al., 2022).

Stress is considered to be a part of students’ academic life due to the internal and external expectations they face (Reddy et al., 2018). Many sources of stress have been identified, including fear of failure, comparison and/or competition with other students, workload, interpersonal difficulties with teachers and/or peers, balancing school life, inadequate resources, and parental pressure (Deb et al., 2015; Reddy et al., 2018; Leslie et al., 2021; Mayya et al., 2022; Perrella et al., 2024). However, the stress experienced by students is not limited to academic stress. It is multifactorial and stems from several factors, including financial situation, romantic life, health, family relationships, and problems experienced by loved ones, in addition to academic stressors (Beiter et al., 2015; Karyotaki et al., 2020). From a cognitive perspective, stress is viewed as an individual process of appraising and managing situations (Lazarus and Folkman, 1984), with coping as a central aspect. Coping refers to cognitive and behavioral efforts to manage external or internal demands that are perceived as taxing on the person’s resources (Lazarus and Folkman, 1984). Although a large number of studies have been conducted and many forms of coping have been proposed, the structure of coping remains unresolved (García-Jiménez et al., 2024). A common distinction is between emotion-focused and problem-focused coping. Problem-focused coping refers to the person’ efforts to change the situation. Emotion-focused coping refers to the efforts the person makes to regulate or control the emotions evoked by stressful situations (Lazarus and Folkman, 1984). Problem-focused coping is considered more adaptive because it is generally associated with positive outcomes such as greater feelings of efficacy and control and has been associated with less depression, whereas emotion-focused coping involves denial and avoidance and has been associated with negative outcomes such as greater distress or depression (Watson and Sinha, 2008; Matud, 2016a; Ben-Zur, 2009). While findings are inconsistent, some studies have found that problem-focused coping is more common in boys (Kaur, 2024), whereas emotion-focused coping is more common in girls (Graves et al., 2021).

Although there is much research on student stress and mental health, to our knowledge, most studies have focused on analyzing such issues in either university or non-university students, but have not analyzed whether there are differences in stress, coping styles, and mental symptoms between university and non-university students. Furthermore, studies generally do not analyze risk and protective factors for students’ mental health, considering their age and educational level, the academic and non-academic stressors they face, their typical stress coping styles, and the importance of self-esteem and social support. Another important shortcoming is that although many studies have analyzed whether there are differences in stress and mental health between male and female students, studies have generally not been conducted from a gender perspective, where, in addition to comparing mean stress and mental health scores between boys and girls, all data are disaggregated by gender. Therefore, the first research question asks whether there are differences between girls and boys and between university and non-university students in terms of sources of stress, coping styles, and mental health. The second research question asks about the importance of age, education, stress, coping styles, self-esteem, and social support on boys’ and girls’ mental symptoms and well-being. The present study follows the World Health Organization’s (WHO) conceptualization of mental health. According to World Health Organization (2022, p. 8), mental health is “a state of mental well-being that enables people to cope with the stresses of life, to realize their abilities, to learn well and work well, and to contribute to their communities. Mental health is an integral component of health and well-being and is more than the absence of mental disorder.” Therefore, in addition to analyzing the relevance of stress to students’ mental symptoms, the relevance of stress to psychological well-being and life satisfaction will also be analyzed. Thus, the aim of this study is to analyze students’ sources of stress and whether there are gender and university/non-university differences in stress, coping styles, mental symptoms, psychological well-being, and life satisfaction. A second aim is to know the relevance of age, education, stress, coping styles as well as self-esteem and social support on mental symptoms, psychological well-being and life satisfaction for student boys and girls.

## 2 Materials and methods

### 2.1 Participants and procedures

The sample was non-probability. It consisted of 1,426 students (872 girls and 554 boys) between the ages of 16 and 26. The mean age for girls was 19.07 years (*SD* = 2.63) and for boys 18.34 years (*SD* = 2.59). 38.8% of the sample (*n* = 553) were in university education and 61.2% (*n* = 873) were in non-university education. Of the non-university students, 56.6% (52.7% of boys and 60.1% of girls) were in high school, 38.5% (42.5% of boys and 34.9% of girls) were in compulsory secondary education, and 4.9% (4.9% of boys and 5% of girls) were in vocational training. Access to the sample was through various university and non-university educational centers located in different Spanish municipalities, which were asked to collaborate in the study. The sample was also accessed through the social network of undergraduate and graduate students in sociology and psychology, who participated in the administration of the tests and received course credit for their participation. An appointment was made with all students who agreed to participate in the study. At this appointment, undergraduate and graduate psychology and sociology students trained in test administration explained to each student the aims of the study and how to complete the tests. All participants who gave informed consent were given an envelope containing the printed questionnaires and instructions on how to complete them. A new appointment was made to collect the completed questionnaires from each participant. In order for the sample to be socio-demographically representative of the population of students aged 16 and over, the following criteria were established in addition to being a student: (1) being between 16 and 26 years old, (2) not having children.

All participants were volunteers and received no financial compensation for their participation in the study. Informed consent was given verbally, so that individuals did not have to sign or leave their personal information on any document. The study was conducted according to the tenets of the Declaration of Helsinki. Participants’ identities were not recorded, and individuals could withdraw at any time. This study is part of a larger investigation on gender and well-being. It was approved by the Animal Research and Welfare Ethics Committee of the University of La Laguna (study approval number 2019–0365).

### 2.2 Measures

#### 2.2.1 Stress

Three measures of stress were collected: (1) Stressful life events. These were assessed using the Life Events Questionnaire (Matud, 1998a). It consists of 27 items asking about the presence of events and changes in the past 12 months in different domains, such as studies, family, friends, romantic relationships, violence and health. In addition, individuals could report any other events that had occurred during this period. Five of the 27 items were directly related to study-related events and/or changes: changing studies, having to leave studies, starting new studies, study-related changes, bullying. Each event was scored as 1 if it occurred and 0 it did not. Thus, higher scores indicate a greater number of stressful events. (2) Chronic stress was measured using the Chronic Stress Questionnaire (Matud, 1998a). This is an open-response instrument that asks participants for information about their current problems, conflicts, and stressors. The importance of each problem is rated from 1 for “not very important” to 3 for “very important.” In the present study, the chronic stress score was obtained by summing the importance scores for each of the problems mentioned. Higher scores indicated more chronic stress. (3) Study dissatisfaction. This was assessed using the student version of the Job Role Satisfaction Questionnaire (Matud, 2016b). This is an open-ended questionnaire consisting of 5 questions about whether the person enjoys his or her studies, whether he or she would have preferred to study elsewhere, whether he or she is thinking about changing, the extent to which his or her studies give him or her a sense of accomplishment, and whether his or her studies make him or her feel good about himself or herself. Responses to each of the open-ended questions were scored quantitatively using a validated code. Higher scores indicate greater dissatisfaction with their studies. For the current sample, the internal consistency (Cronbach’s alpha) of the 5 items was 0.71.

#### 2.2.2 Coping styles

Stress coping styles were assessed using the Spanish version of the Coping Styles Questionnaire developed by Roger et al. (1993). It consists of 46 items measuring typical coping with stress and is structured into three factors: rational coping style, consisting of 15 items (e.g., “Use my past experience to try to deal with situation”); emotional coping style, consisting of 16 items (e.g., “Feel helpless -there’s nothing you can do about it”); and detachment/avoidance coping style, consisting of 15 items (e.g., “Try to think about or do something else”). The response scale is a 4-point Likert scale ranging from 0 (never) to 3 (always). Higher scores indicate greater coping style. For the current sample, Cronbach’s alpha was 0.80 for the rational coping style, 0.82 for the emotional coping style, and 0.75 for the detachment/avoidance coping style.

#### 2.2.3 Mental symptoms

Mental symptoms were assessed using the Spanish version (Goldberg et al., 1996) of the 28-item form of the Goldberg General Health Questionnaire (GHQ-28) (Goldberg and Hillier, 1979). This is a self-administered screening test consisting of 28 items structured into four scales that are not independent. Each scale consists of 7 items measuring somatic symptoms (e.g., “Been getting any pains in your head”), anxiety and insomnia (e.g., “Lost much sleep over worry”), social dysfunction (e.g., “Been taking longer over the things you do?”), and severe depression symptoms (e.g., “Felt that life is entirely hopeless?”). Items are scored on a Likert scale ranging from 0 (less than usual) to 3 (much more than usual). Higher scores indicate more symptoms. For the current sample, Cronbach’s alpha was 0.79 for the somatic symptom scale, 0.87 for anxiety and insomnia, 0.72 for social dysfunction, and 0.89 for severe depression. The Cronbach’s alpha for the 28 items of the questionnaire was 0.90.

#### 2.2.4 Psychological well-being

Psychological well-being was assessed using the Spanish version of the Ryff Psychological Well-Being Scale (van Dierendonck et al., 2008). This scale measures eudaemonic well-being, a state of positive human functioning that emphasizes the importance of personal growth and development (van Dierendonck and Lam, 2023). This version consists of 38 items divided into six scales and a second-order latent construct of psychological well-being (van Dierendonck et al., 2008). The six scales are: self-acceptance, consisting of 6 items (e.g., “In general, I feel confident and positive about myself”) with an internal consistency (Cronbach’s alpha) of 0.84 for the current sample; positive relationships, consisting of 6 items (e.g., “Most people see me as loving and affectionate”) with a Cronbach’s alpha of 0.79; autonomy, consisting of 8 items (e.g., “My decision are not usually influenced by what everyone else is doing”) with a Cronbach’s alpha of 0.75; environmental mastery, consisting of 6 items (e.g., “In general, I feel I am in charge of the situation in which I live”) with a Cronbach’s alpha of 0.64; purpose in life, consisting of 6 items (e.g., “I have a sense of direction and purpose in life”) with a Cronbach’s alpha of 0.81; and personal growth, consisting of 6 items with a Cronbach’s alpha of 0.74 (e.g., “I have a sense that I have developed a lot as a person over time”). The scale response is a 6-point Likert scale ranging from 1 (strongly disagree) to 6 (strongly agree), with higher scores indicating greater psychological well-being. For the current sample, the Cronbach’s alpha of the 38 items comprising the latent construct of psychological well-being was 0.92.

#### 2.2.5 Life satisfaction

Life satisfaction was measured using the Satisfaction with Life Scale (SWLS) (Diener et al., 1985). The SWLS is a 5-item scale that assesses overall satisfaction with life, which is considered the cognitive component of subjective well-being. Sample items include “In most ways my life is close to my ideal” and “I am satisfied with life.” The response scale is a 7-point Likert scale ranging from 1 (strongly disagree) to 7 (strongly agree). Higher scores indicate greater life satisfaction. For the current sample, Cronbach’s alpha was 0.82.

#### 2.2.6 Self-esteem

The Spanish version of the York Self-esteem Inventory (Matud et al., 2003b) was used to measure self-esteem. This inventory consists of 51 items that assess global self-esteem. It covers different self-domains such as personal, interpersonal, family, and achievement. Sample items include “I feel content with the way I am” and “My friends consider me very reliable.” The response scale is a 4-point Likert scale ranging from never, scored 0, to always, scored 3. Higher scores indicate higher self-esteem. For the current sample, Cronbach’s alpha was 0.94.

#### 2.2.7 Social support

Social support was measured using the Social Support Scale (SSS) (Matud, 1998b). The SSS is a scale developed and validated for the Spanish general population (Matud et al., 2003a). It consists of 12 items that assess perceived social support in the emotional, instrumental, and informational domains. Sample items include “Someone who comforts you when you are upset” and “Someone who lends you money when you have economic problems.” The response is a 4-point Likert scale ranging from never, scored 0, to always, scored 3. Higher scores indicate greater perceived social support. For the current sample, Cronbach’s alpha was 0.88.

### 2.3 Statistical analysis

Nine analyses of variance (ANOVAs) were conducted to answer the first research question, which asked whether there were differences between girls and boys and between university and non-university students in terms of sources of stress, coping styles, and mental health. In all analyses, the factors were gender (boys, girls) and education (non-university, university), and the dependent variables were measures of stress, coping styles, mental symptoms, psychological well-being, and life satisfaction. Analysis of the ANOVA assumption of homogeneity of variance showed that the variance was homogeneous for all variables, except study dissatisfaction and mental symptoms. Therefore, comparisons on these variables are also analyzed by nonparametric tests using Welch’s test and Brown-Forsythe test. For both variables, *post hoc* comparisons were analyzed using the Games-Howell test. This test does not assume homogeneity of variance. For the remaining variables, *Post hoc* comparisons were performed using Scheffé’s adjustment.

Bivariate correlation and hierarchical multiple regression analyses were used to answer the second research question, which asked about the relevance of age, education, stress, coping styles, self-esteem, and social support on boys’ and girls’ mental symptoms and well-being. Bivariate associations were calculated using Pearson’s correlation coefficient, except for education, which was calculated using Spearman’s Rho because it is an ordinal variable. In each regression analysis, age was included as a continuous variable and education as an ordinal variable with 7 levels ranging from 1 for compulsory secondary education to 7 for 5 years of university education in the first step (Model 1). Model 2 added stress scores. Model 3 added coping styles scores. Model 4 added self-esteem and social support scores. The dependent variables were total mental symptom score (calculated by summing the 28 items of the GHQ-28) in the first regression analyses. Total psychological well-being score (the second-order latent construct of psychological well-being) in the second regression analyses. And the life satisfaction score in the third regression analyses. The mental symptom score was highly skewed, with predominantly low scores. Therefore, a logarithmic transformation was applied, following the recommendations of Tabachnick and Fidell (2019).

Correlation and multiple regression analyses were performed independently for the groups of boys and girls. Statistical analyses were performed with IBM SPSS Statistics for Windows, version 22.0.

## 3 Results

First, we will present the results of the association for boys and girls between the three measures of stress used in this study: the number of stressful events and changes experienced in the past 12 months, chronic stress, and study dissatisfaction. In addition, the results of the association for boys and girls between the three measures of stress and stress coping styles will be analyzed. This will deepen our understanding of the stress measures used and the association between stress and coping styles. We will then present the results of the differences in stress and coping between girls and boys and between university and non-university students. Next, we present the results of the bivariate associations of stress and coping with the other study variables. Finally, we will present the results of the regression analyses of the relevance of age, education, stress, coping styles, self-esteem, and social support in predicting of mental health symptoms, psychological well-being, and life satisfaction for girls and boys.

Intercorrelations for the three stress measures indicated that the number of stressful events and changes in the past 12 months had a statistically significant (*p* < 0.001) association with chronic stress for both genders (*r* = 0.30 for girls and *r* = 0.22 for boys). The number of stressful events was also statistically significantly associated with study dissatisfaction for girls (*r* = 0.15, *p* < 0.001), but not for boys (*r* = 0.06, *p* = 0.16). And chronic stress was independent of study dissatisfaction for girls (*r* = 0.04, *p* = 0.26) and for boys (*r* = 0.05, *p* = 0.26). All this suggests that the three stress measures used assess different types of stress, although a greater number of stressful events tends to be associated with greater chronic stress. The analysis of differences between girls and boys in the association between the number of stressful events in the past 12 months and chronic stress (*z* = 1.56, *p* = 0.12), study dissatisfaction (*z* = 1.66, *p* = 0.09), and between chronic stress and study dissatisfaction (*z* = 0.18, *p* = 0.85) did not reveal statistically significant differences.

Correlational analyses were conducted to determine whether experienced stress was associated with boys’ and girls’ stress coping styles (see Table 1). There were some statistically significant correlations, although the effect size was small. For both genders, greater stress was associated with a more emotional coping style, and greater study dissatisfaction was associated with a less rational coping style. Analysis of differences between girls and boys in the association of stress measures with stress coping styles revealed statistically significant differences in the association of chronic stress with rational coping style (*z* = 2.39, *p* = 0.02). There were also statistically significant differences in the association of study dissatisfaction with emotional coping style (*z* = 2.48, *p* = 0.01) and with detachment/avoidance (*z* = 2.21, *p* = 0.03). Only for boys was greater chronic stress associated with greater rational coping style, although the effect size was very small. The association between study dissatisfaction and emotional coping was stronger for girls than for boys. And only for girls was greater study dissatisfaction associated with greater detachment/avoidance coping style, although the effect size was very small.

**Table 1:** Correlations between stress and coping styles disaggregated by gender.

Variable	Girls stress coping styles			Boys stress coping styles		
Stress	Emotional	Rational	Detachment/avoidance	Emotional	Rational	Detachment/avoidance
Number of stressful events	0.21***	−0.03	0.10**	0.11*	0.06	0.01
Chronic stress	0.22***	−0.03	−0.02	0.13**	0.10*	−0.08
Study dissatisfaction	0.25***	−0.23***	0.10**	0.12**	−0.16***	−0.02

*p < 0.05; **p < 0.01; ***p < 0.001.

### 3.1 Differences in stress and in stress coping styles between girls and boys and between university and non-university students

Table 2 shows the main results of the ANOVAs with gender (boys, girls) and education (non-university, university) as factors and each of the stress, stress coping styles, and mental health scores as dependent variables. As can be seen, there were no statistically significant gender × education interactions on any of the three stress measures. Also, there were no statistically significant differences between girls and boys or between university and non-university students in the number of stressful events. The ANOVA with chronic stress as the dependent variable revealed that only the main effect of education was statistically significant, although the effect size was small. As shown in Table 2, university students had more chronic stress than non-university students. When the dependent variable was study dissatisfaction, the main effects of gender and education were statistically significant. Welch’s test and Brown-Forsythe test also showed statistically significant differences between the groups (*p* < 0.000). *Post hoc* analyses using Games-Howell’s adjustment revealed that non-university girls and boys were more dissatisfied with their studies than university girls and boys (*p* < 0.000). In addition, non-university boys were more dissatisfied with their studies than non-university girls (*p* = 0.02). And university boys were more dissatisfied with their studies than university girls (*p* = 0.032).

**Table 2:** Means (*M*), standard deviations (*SD*) and two-way ANOVA statistics for stress, coping styles, mental symptoms, psychological well-being and life satisfaction.

Variable	Boys		Girls		ANOVA		
	*M*	*SD*	*M*	*SD*	Effect	*F Ratio*	η_p_^2^
Number of stressful events							
Non-university	2.38	2.15	2.39	2.05	Gender	0.56	0.000
University	2.67	2.23	2.48	1.91	Education	2.35	0.002
Interaction Gender × Education					G × E	0.68	0.000
Chronic stress							
Non-university	3.52	3.44	3.85	3.78	Gender	1.10	0.001
University	5.08	3.42	5.20	3.45	Education	47.49***	0.032
Interaction Gender × Education					G × E	0.23	0.000
Study dissatisfaction							
Non-university	5.33	3.96	4.58	3.63	Gender	13.02***	0.009
University	3.43	2.56	2.75	2.38	Education	89.06***	0.059
Interaction Gender × Education					G × E	0.34	0.000
Emotional coping style							
Non-university	17.20	6.55	16.94	6.63	Gender	0.36	0.000
University	14.81	6.59	15.54	6.69	Education	23.33***	0.016
Interaction Gender × Education					G × E	1.63	0.001
Rational coping style							
Non-university	24.45	6.08	24.14	6.29	Gender	11.76**	0.008
University	28.47	5.76	26.34	5.73	Education	76.00***	0.051
Interaction Gender × Education					G × E	6.44*	0.005
Detachment/avoidance coping style							
Non-university	18.43	5.78	16.99	5.40	Gender	30.66***	0.021
University	17.25	5.12	15.04	5.62	Education	22.44***	0.016
Interaction Gender × Education					G × E	1.36	0.001
Mental symptoms							
Non-university	21.75	12.12	23.42	12.38	Gender	7.57**	0.005
University	18.08	10.35	20.19	10.43	Education	25.27***	0.017
Interaction Gender × Education					G × E	0.10	0.000
Psychological well-being							
Non-university	161.36	23.30	168.00	23.27	Gender	10.03**	0.007
University	174.01	21.78	175.96	22.36	Education	57.71***	0.039
Interaction Gender × Education					G × E	2.98	0.002
Life satisfaction							
Non-university	23.68	5.91	24.38	5.95	Gender	4.63*	0.003
University	24.52	5.51	25.32	5.86	Education	6.54*	0.005
Interaction Gender × Education					G × E	0.02	0.000

*p < 0.05; **p < 0.01; ***p < 0.001.

**Figure 1:**
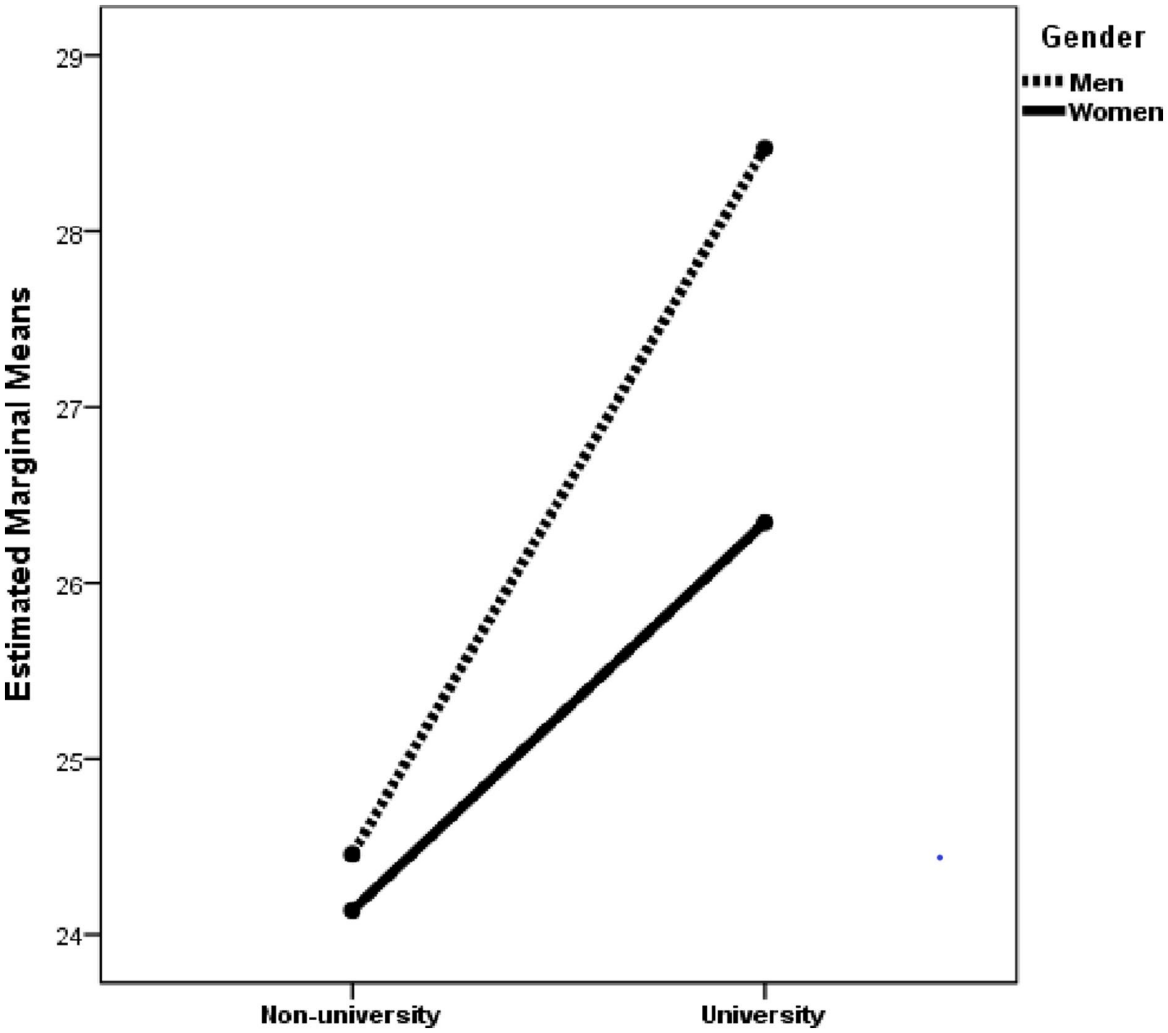
Changes in rational coping style as a function of gender and education.

ANOVAs in which the dependent variable was stress coping styles indicated that the gender × education interaction was statistically significant when the dependent variable was rational coping style (see Table 2 and Figure 1). *Post hoc* analyses with Scheffe adjustment revealed that there were no statistically significant differences (*p* = 0.89) between boys (*M* = 24.45, *SD* = 6.08) and girls (*M* = 24.14, *SD* = 6.29) with non-university education. However, university boys scored higher (*p* = 0.004) on the rational coping style (*M* = 28.47, *SD* = 5.76) than university girls (*M* = 26.34, *SD* = 5.73). In addition, university girls and boys had higher rational coping style scores than non-university girls (*p* < 0.001) and boys (*p* < 0.001).

When the dependent variable was emotional coping style, only the main effect of education was found to be statistically significant. As can be seen in Table 1, university students had a lower emotional coping style than non-university students. When the dependent variable was detachment/avoidance coping style, the main effects of gender and education were statistically significant. University girls had a lower detachment/avoidance stress coping style than the other groups, and non-university girls had a lower detachment/avoidance stress coping style than non-university boys. When considering mental symptoms as the dependent variable, the main effects of education and gender were statistically significant. Welch’s test and Brown-Forsythe test also showed statistically significant differences between the groups (*p* < 0.000). *Post hoc* analyses with Games-Howell’s adjustment revealed that non-university girls had more mental symptoms than university girls and boys (*p* < 0.000). In addition, non-university boys had more mental symptoms than university boys (*p* = 0.003). When psychological well-being was considered as the dependent variable, the main effects of education and gender were statistically significant, although the effect size of gender was much smaller than that of education. Non-university boys had lower psychological well-being than the other groups. In addition, non-university girls had lower psychological well-being than university girls. When life satisfaction was considered as the dependent variable, the main effects of gender and education were statistically significant, although the effect sizes were very small. *Post hoc* analyses with Scheffe adjustment revealed that there were only statistically significant differences (*p* = 0.001) between non-university boys and university girls. As shown in Table 2, non-university boys had lower life satisfaction than university girls.

Analysis of events and/or changes in the past 12 months revealed that most participants (82.5%) reported experiencing one or more events and/or changes. The percentages were 86.4% for university girls, 81.8% for non-university girls, 81.7% for university boys, and 79.6% for non-university boys. The differences were not statistically significant, χ^2^ (3, *N* = 1,426) = 6.87, *p* = 0.076. Table 3 shows the most common non-academic and academic events and/or changes experienced in the past 12 months. It also shows the percentage of each group that experienced each event. As can be seen, the most reported event was family arguments, an event for which there were statistically significant differences between the groups. More than a third of non-university girls (39%), but less than a quarter of university boys (21.8%) reported this event. The next most common event was the serious illness of a family member, reported by 27.6% of the total sample, with no statistically significant differences between the groups. The next most common event was the beginning of a romantic relationship. This event was reported by more than a quarter of the sample, except for university girls, where only 19.2% reported it. The next most common event was the breakup of a romantic relationship, followed by the death of a family member. There were no statistically significant differences between the groups for these events. The next most common event was changes in relationships with parents. This was reported by a quarter of non-university girls and a fifth of non-university boys and was less common among university girls and boys. There were also statistically significant differences between the groups in moving house. This was reported by a quarter of university boys and a fifth of university girls, but much less frequently by non-university girls and boys. The next most common event was serious arguments with their partner, reported by almost 10% of the sample. The remaining life events were less common, reported by less than 8% of the total sample.

**Table 3:** Most common non-academic and academic events and/or changes experienced in the past 12 months.

Event	Non-university boys	University boys	Non-university girls	University girls	*χ*^2^	*p*
Non-academic events						
Family arguments	32.8%	21.8%	39.0%	32.6%	15.24	0.002
Serious illness of a family member	23.3%	28.9%	27.8%	31.1%	6.52	0.089
Beginning a romantic relationship	26.5%	28.9%	29.5%	19.2%	13.42	0.004
Breakup of a romantic relationship	26.2%	32.4%	25.2%	22.1%	6.14	0.105
Death of a family member	22.6%	14.8%	24.9%	22.6%	6.41	0.093
Changes in relationships with their parents	20.9%	16.9%	25.2%	16.3%	11.74	0.008
Moving house	12.1%	24.6%	11.3%	19.7%	24.69	<0.001
Serious arguments with their partner	10.2%	9.9%	8.7%	10.9%	1.32	0.724
Academic events						
Starting new studies	3.4%	10.6%	3.3%	9.7%	26.93	< 0.001
Change in study conditions	3.2%	6.3%	1.1%	3.2%	11.89	0.008
Switching studies	1.2%	6.3%	1.3%	3.4%	16.13	0.001
Dropping out	0.7%	3.5%	1.7%	1.9%	5.35	0.148
Bulling	0.5%	0.0%	0.9%	0.5%	1.66	0.646

At least one academic-related event was reported by 9.8% of the total sample. It occurred in 16.2% of university boys, 13.1% of university girls, 7.4% of non-university girls, and 7.0% of non-university boys. The differences in percentages were statistically significant, χ^2^ (3, *N* = 1,426) = 18.36, *p* < 0.001. Table 3 also shows, from highest to lowest frequency, the percentage of individuals in each group who experienced each type of academic-related event or change assessed. As can be seen, there were statistically significant differences for all types of events except dropping out and bullying. Bullying was the least common event, reported by only two non-university boys, four non-university girls, and two university girls. Starting new studies was the most common academic event, reported much more frequently by both university boys and girls. Changing study conditions was reported most frequently by university boys (6.3%) and very rarely by non-university girls (1.1%). Switching was also more common among university students than non-university students and affected more boys (6.3%) than girls (3.4%).

### 3.2 Bivariate associations of stress and stress coping styles with study variables for girls and boys

Table 4 shows the results of the bivariate correlations between stress and the study variables, disaggregated by gender. For both genders, older age and higher education were associated with more chronic stress and less study dissatisfaction. More stressful events, chronic stress, and study dissatisfaction were associated with more mental symptoms, lower life satisfaction, lower self-esteem, lower social support, and lower self-acceptance. In addition, greater study dissatisfaction was associated with less environmental mastery, purpose in life, and personal growth. Analysis of statistically significant differences between girls and boys in the association between stress measures and the other study variables revealed the existence of statistically significant gender differences in the association between the number of stressful events and age (*z* = 2.60, *p* < 0.01) and between study dissatisfaction and education (*z* = −2.51, *p* = 0.01). As shown in Table 4, age was associated with a greater number of stressful events for boys but not for girls, while study dissatisfaction was more strongly associated with a lower level of education for girls than for boys. There were also statistically significant differences in the association between chronic stress and somatic symptoms (*z* = 2.09, *p* = 0.04); and between study dissatisfaction and somatic symptoms (*z* = 2.07, *p* = 0.04) and severe depression symptoms (*z* = 3.45, *p* < 0.001). The association between these measures of stress and mental symptoms was stronger for girls than for boys. When analyzing differences between girls and boys in the association of stress with well-being, self-esteem, and social support, statistically significant differences were found only for the association between number of stressful events and environmental mastery (*z* = 2.05, *p* = 0.04), between chronic stress and autonomy (*z* = 2.58, *p* = 0.01) and personal growth (*z* = −2.76, *p* < 0.01), and between study dissatisfaction with positive relationships (*z* = 2.62, *p* < 0.01) and self-esteem (*z* = 2.50, *p* = 0.01). For girls, more chronic stress was associated with less autonomy and less environmental mastery. For boys, more chronic stress was associated with more personal growth. The association between greater study dissatisfaction and lower self-esteem was stronger for girls than for boys. It was only for the girls that study dissatisfaction was associated with fewer positive relationships.

**Table 4:** Correlations between stress and study variables disaggregated by gender.

	Girls			Boys		
Variable	Number of stressful events	Chronic stress	Study dissatisfaction	Number of stressful events	Chronic stress	Study dissatisfaction
Age	0.02	0.20***	−0.27***	0.16***	0.20***	−0.19***
Education ^a^	0.03	0.23***	−0.28***	0.13**	0.26***	−0.15***
Somatic symptoms	0.25***	0.23***	0.20***	0.21***	0.12**	0.09*
Anxiety and insomnia	0.25***	0.25***	0.20***	0.25***	0.21***	0.12**
Severe depression	0.22***	0.15***	0.29***	0.15***	0.11*	0.11*
Social dysfunction	0.10***	0.18***	0.20***	0.05	0.12**	0.17***
Self-acceptance	−0.15***	−0.19***	−0.33***	−0.11***	−0.15***	−0.26***
Positive relationships	−0.06	−0.11**	−0.20***	−0.05	−0.08	−0.06
Autonomy	−0.06	−0.10**	−0.13***	−0.06	0.04	−0.05
Environmental mastery	−0.18***	−0.13***	−0.34***	−0.07	−0.05	−0.33***
Purpose in life	−0.11**	−0.10**	−0.40***	−0.03	−0.05	−0.29***
Personal growth	−0.08*	−0.03	−0.26***	−0.01	0.12**	−0.17***
Life Satisfaction	−0.17***	−0.24***	−0.31***	−0.15**	−0.20***	−0.26***
Self-esteem	−0.16***	−0.21***	−0.27***	−0.10*	−0.12**	−0.14**
Social support	−0.18***	−0.12***	−0.23***	−0.10*	−0.11**	−0.13**

^a^ Coefficient calculated using Spearman’s Rho. *p < 0.05; **p < 0.01; ***p < 0.001.

Correlations between boys’ and girls’ stress coping styles and the study variables are presented in Table 5. In general, and for both genders, higher age and education were associated with more rational and less emotional and detachment/avoidance stress coping styles. For both genders, a higher emotional stress coping style was associated with more mental symptoms, lower well-being, lower self-esteem, and lower social support. A higher rational stress coping style was associated with fewer mental symptoms and higher well-being, self-esteem and social support. And a more detachment/avoidance coping style was associated with more symptoms of severe depression and anxiety and insomnia, and with lower self-esteem.

**Table 5:** Correlations between stress coping styles and study variables disaggregated by gender.

	Girls			Boys		
Variable	Emotional	Rational	Detachment/avoidance	Emotional	Rational	Detachment/avoidance
Age	−0.10**	0.14***	−0.19***	−0.16***	0.29***	−0.12**
Education ^a^	−0.10**	0.21***	−0.16***	−0.09*	0.25***	−0.06
Somatic symptoms	0.41***	−0.18***	0.04	0.44***	−0.12**	0.17***
Anxiety and insomnia	0.50***	−0.16***	0.10**	0.48***	−0.06	0.11**
Severe depression	0.54***	−0.26***	0.15***	0.57***	−0.23***	0.23***
Social dysfunction	0.39***	−0.23***	0.04	0.33***	−0.21***	0.07
Self-acceptance	−0.56***	0.44***	−0.02	−0.51***	0.42***	−0.08
Positive relationships	−0.42***	0.24***	−0.08*	−0.41***	0.28***	−0.17***
Autonomy	−0.34***	0.31***	−0.05	−0.36***	0.33***	−0.18***
Environmental mastery	−0.49***	0.45***	−0.07*	−0.41***	0.42***	−0.13**
Purpose in life	−0.41***	0.44***	−0.05	−0.31***	0.41***	−0.09*
Personal growth	−0.32***	0.46***	−0.11**	−0.31***	0.41***	−0.15***
Life Satisfaction	−0.40***	0.33***	−0.05	−0.36***	0.23***	−0.04
Self-esteem	−0.67***	0.45***	−0.10**	−0.70***	0.43***	−0.24***
Social support	−0.35***	0.25***	−0.05	−0.28***	0.18***	−0.03

^a^ Coefficient calculated using Spearman’s Rho. *p < 0.05; **p < 0.01; ***p < 0.001.

Analysis of statistically significant differences between girls and boys in the association between stress coping styles and the study variables revealed the existence of statistically significant gender differences in the association between emotional coping style and purpose in life (*z* = 2.11, *p* = 0.03); between rational coping style and age (*z* = 2.89, *p* < 0.01) and life satisfaction (*z* = 1.99, *p* = 0.04); and between detachment/avoidance coping and somatic symptoms (*z* = 2.42, *p* = 0.02), autonomy (*z* = 2.42, *p* = 0.02), and self-esteem (*z* = 2.65, *p* < 0.01). The strength of the association between a more emotional coping style and less purpose in life was greater for girls than for boys. The association between older age and more rational coping was stronger for boys than for girls. The strength of the association between rational coping style and life satisfaction was greater for girls than for boys. The more detachment/avoidance coping style was associated with more somatic symptoms and less autonomy only for boys, while the association between this coping style and lower self-esteem was stronger for boys than for girls.

### 3.3 Relevance of age, education, stress, coping styles, self-esteem and social support in predicting mental symptoms, psychological well-being and life satisfaction for girls and boys

Table 6 presents the main results of the hierarchical regression analysis predicting the logarithm of mental symptoms for girls and boys. Model 1 was statistically significant for girls only. The three stress measures entered in step 2 (Model 2) explained 8.8% of the variance for boys and 15% for girls. The standardized coefficients (*β*) for all three stress measures were statistically significant. The coping styles added in step 3 (Model 3) explained an additional 25.6% of the variance for boys and 21.5% for girls. The addition of self-esteem and social support in step 4 (Model 4) explained an additional 1.9% of the variance for boys and 1.8% for girls. Although the magnitude of the β coefficients for the stress measures decreased when coping styles were added in step 3, and the β coefficient for study dissatisfaction was no longer statistically significant for the boys’ sample, in the final model with all variables in the equation, more stressful events and more chronic stress predicted more mental symptoms for both boys and girls. However, for both genders the main predictor of mental symptoms was a more emotional coping style, followed by lower self-esteem. In addition, for girls, more study dissatisfaction predicted more mental symptoms. The total variance explained was 35.8% for boys and 38.6% for girls.

**Table 6:** Summary of hierarchical regression with mental symptoms (logarithmic transformation) as the dependent variable for girls and boys.

		Boys			Girls			
	Model 1	Model 2	Model 3	Model 4	Model 1	Model 2	Model 3	Model 4
	*β*	*β*	*β*	*β*	*β*	*β*	*β*	*β*
Age	−0.08	−0.11	0.01	0.00	0.01	−0.01	0.01	0.01
Education	−0.00	−0.02	−0.00	0.00	−0.11	−0.07	−0.04	−0.03
Number of stressful events		0.18***	0.13***	0.12**		0.17***	0.12***	0.11***
Chronic stress		0.17***	0.11**	0.09*		0.21***	0.11***	0.10**
Study dissatisfaction		0.10*	0.05	0.04		0.21***	0.10**	0.09**
Emotional coping style			0.49***	0.35***			0.47***	0.36***
Rational coping style			−0.13***	−0.05			−0.11***	−0.04
Detachment/avoidance coping style			0.02	0.01			−0.02	−0.03
Self-esteem				−0.20***				−0.17***
Social support				−0.06				−0.07*
*R*^2^ change	0.006	0.088***	0.256***	0.019***	0.010**	0.150***	0.215***	0.018***
Adj. *R*^2^	0.003	0.086	0.341	0.358	0.007	0.155	0.369	0.386

β, standardized regression coefficient; Adj. *R*^2^, percentage of explained variance; *p < 0.05, **p < 0.01, ***p < 0.001.

Table 7 shows the main results of the hierarchical regression analysis for predicting psychological well-being for girls and boys. Age and education, included in Model 1, explained 6.0% of the variance for boys and 3.1% of the variance for girls. The three stress measures included in Model 2 explained an additional 5.3% of the variance for boys and 13.3% for girls. However, only the *β* coefficient for study dissatisfaction was statistically significant for boys, whereas the β coefficient for chronic stress was also statistically significant for girls. The coping styles added in Model 3 explained an additional 38.4% of the variance for boys and 35.2% for girls. The addition of self-esteem and social support in Model 4 explained an additional 12.6% of the variance for boys and 17.9% for girls. While the magnitude of the β coefficients for the stress measures decreased as coping styles were added in Model 3, and the β coefficient for chronic stress was no longer statistically significant for the girls’ sample, in the final model with all variables in the equation, less study dissatisfaction predicted greater well-being for both boys and girls. For both genders, the most important predictor of greater psychological well-being was higher self-esteem. For boys, other statistically significant predictors of greater well-being were more rational coping style, more social support, more education, younger age, and less detachment/avoidance coping style. For girls, the second most important predictor of greater psychological well-being was greater social support, followed by a more rational coping style. In addition, younger age, higher education, and a less emotional coping style also predicted greater psychological well-being for girls. The percentage of total variance explained was 62% for boys and 69.3% for girls.

**Table 7:** Summary of hierarchical regression with psychological well-being as the dependent variable for girls and boys.

		Boys			Girls			
	Model 1	Model 2	Model 3	Model 4	Model 1	Model 2	Model 3	Model 4
	*β*	*β*	*β*	*β*	*β*	*β*	*β*	*β*
Age	0.06	0.05	−0.12*	−0.11*	−0.10	–0.11*	−0.12**	−0.10**
Education	0.21**	0.20**	0.15**	0.14**	0.26***	0.20***	0.09*	0.07*
Number of stressful events		−0.07	−0.04	−0.01		−0.05	−0.00	0.02
Chronic stress		−0.07	−0.04	0.01		−0.14***	−0.04	0.01
Study dissatisfaction		−0.19***	−0.12***	−0.10***		−0.32***	−0.16***	−0.12***
Emotional coping style			−0.40***	−0.06			−0.42***	−0.07**
Rational coping style			0.46***	0.24***			0.40***	0.19***
Detachment/avoidance coping style			−0.10**	−0.07*			−0.06*	−0.03
Self-esteem				0.48***				0.52***
Social support				0.20***				0.23***
*R*^2^ change	0.064***	0.053***	0.384***	0.126***	0.033**	0.133***	0.352***	0.179***
Adj. *R*^2^	0.060	0.108	0.493	0.620	0.031	0.161	0.514	0.693

β, Standardized regression coefficient; Adj. *R*^2^, percentage of explained variance; *p < 0.05, **p < 0.01, ***p < 0.001.

Table 8 shows the main results of the hierarchical regression analysis predicting life satisfaction for girls and boys. The sociodemographic variables included in Model 1 were statistically significant only for girls. They predicted 2.3% of the variance. The three stress measures entered in Model 2 explained 11.1% of the variance for boys and 14.7% for girls. The β coefficients of the three stress measures were statistically significant, although the magnitude of the β coefficient for number of stressful events was much smaller. The coping styles added in Model 3 explained an additional 13.4% of the variance for boys and 13.2% for girls. The addition of self-esteem and social support in Model 4 explained an additional 9.8% of the variance for boys and 9.4% for girls. In the final model with all variables in the regression equation, lower study dissatisfaction and lower chronic stress predicted higher life satisfaction for both genders. However, the main predictor of higher life satisfaction was higher self-esteem, followed by higher social support. Another significant predictor of higher life satisfaction for both boys and girls was younger age. In addition, for girls, higher education and a more detached/avoidant stress coping style also predicted greater life satisfaction. The total variance explained was 33.8% for boys and 39.3% for girls.

**Table 8:** Summary of hierarchical regression with life satisfaction as the dependent variable for girls and boys.

		Boys			Girls			
	Model 1	Model 2	Model 3	Model 4	Model 1	Model 2	Model 3	Model 4
	β	β	β	β	β	β	β	β
Age	−0.09	−0.10	−0.19**	−0.17**	−0.24***	−0.24***	−0.23***	−0.22**
Education	0.13	0.14*	0.12	0.10	0.27***	0.22***	0.17**	0.16**
Number of stressful events		−0.09*	−0.06	−0.04		−0.07*	−0.04	−0.02
Chronic stress		−0.18***	−0.14***	−0.10**		−0.20***	−0.14***	−0.11***
Study dissatisfaction		−0.24***	−0.19***	−0.17***		−0.29***	−0.20***	−0.17***
Emotional coping style			−0.31***	−0.06			−0.29***	−0.06
Rational coping style			0.21***	0.05			0.20***	0.06
Detachment/ avoidance coping style			0.03	0.04			0.08*	0.09**
Self-esteem				0.31***				0.31***
Social support				0.25***				0.23***
*R*^2^ change	0.006	0.111***	0.134***	0.098***	0.025***	0.147***	0.132***	0.094***
Adj. *R*^2^	0.003	0.110	0.241	0.338	0.023	0.168	0.299	0.393

β, standardized regression coefficient; Adj. *R*^2^, percentage of explained variance; *p < 0.05, **p < 0.01, ***p < 0.001.

## 4 Discussion

This study, conducted with a sample of 1,436 students aged 16–26, included three measures of stress: the number of stressful academic and non-academic events and changes experienced in the past 12 months, chronic stress, and stress due to study dissatisfaction. Results showed that most students (over 80%) reported experiencing one or more events and/or changes in the past 12 months, consistent with previous literature highlighting that these are changing times (Auerbach et al., 2018; Blakemore, 2019). Analysis of the most frequent sources of stress among students revealed that family arguments were the most common, reported by a third of the sample, although this was more common among non-university girls (39%) and less common among university boys (21.8%). Starting a romantic relationship was a common event, although its frequency was lower among university girls (19.2%) than among non-university girls (29.5%), a percentage very similar to that of university boys (28.9%). The reasons for this are not known. It could be that university girls were more focused and prioritized their studies, as they were the group that showed less dissatisfaction with their studies. This is a hypothesis that should be tested in future studies. The breakup of a romantic relationship was reported by a quarter of the sample. Changes in relationships with parents were also common, although more so for non-university students (25.2% of girls and 20.9% of boys) than for university students (16.9% of boys and 16.3% of girls). Less common were academic-related events, which were reported by 10% of the sample and were more common among university boys (16.2%) and girls (13.1%) than among non-university students (7.4% of girls and 7% of boys). In line with the arguments of other authors (Beiter et al., 2015; Karyotaki et al., 2020), the results of the present work indicate that the sources of student stress are not limited to academic stress, but that other factors are also relevant, among which family and romantic relationships stand out.

The first research question asked whether there are differences between boys and girls and between university and non-university students in sources of stress, coping styles, and mental health. The results of the ANOVAs showed that university students had more chronic stress than non-university students, but less study dissatisfaction, which was greater for boys than for girls. There were no statistically significant differences in the number of stressful events experienced according to gender or education. All this points to the complexity of stress in students. And, although there were some differences according to gender, being a university student or not had greater differentiating power, although the effect of age on these differences cannot be excluded.

The analysis of the relevance of gender and education on mental symptoms showed that although there were statistically significant differences as a function of gender and education, education explained much more variance than gender, with more symptoms in non-university students than in university students. The presence of more mental health symptoms in girls compared to boys is a finding that has been reported in previous literature (Auerbach et al., 2018; Derdikman-Eiron et al., 2011; Twenge et al., 2019; Hermann et al., 2024). In the present study, although the mean scores of mental symptoms were slightly higher in girls than in boys, such differences were not statistically significant when comparing girls and boys of the same educational level. When the scores for psychological well-being were considered as the dependent variable, it was also found that education had a greater differentiating power than gender. Non-university boys had lower well-being than the other groups, and non-university girls had lower well-being than university girls. In addition, university girls had higher life satisfaction than non-university boys.

Analysis of the relevance of gender and education in coping styles revealed a statistically significant interaction gender × education in the rational coping style. Although there were no differences in this coping style between boys and girls who did not have a university education, there were differences among university students, with university boys having a more rational stress coping style than university girls. Furthermore, university boys and girls had a more rational coping style than non-university boys and girls. Statistically significant differences were found only for emotional coping as a function of education. This coping style was less common among university students than among non-university students. Boys were more likely than girls to report detachment/avoidance coping. This coping style was also more common among non-university students than among university students. Taken together, the results of the present study suggest that non-university students have less healthy coping styles. These healthier stress coping styles among university students may explain why they have fewer mental symptoms and greater well-being than non-university students, despite having more chronic and academic stress. The greater well-being and fewer mental symptoms found in university students compared to non-university students in the present study would also be a consequence of the fat that students with better mental health and well-being, as well as those with healthier stress coping styles, are the ones who are more likely to continue their studies and reach university, whereas those with more mental symptoms and less healthy coping styles are at greater risk of dropping out of school. These are hypotheses to be tested in future work. These results are consistent with the approach that universities play an important role in shaping the intellectual growth and personal development of students (Orlova and Shevchenko, 2023).

The second research question asked about the importance of age, education, stress, coping styles, self-esteem and social support on mental symptoms and well-being in boys and girls. The results of the present study showed that more stress was associated with more mental symptoms, findings that are consistent with previous research (Deb et al., 2015; Ribeiro et al., 2018; Jafflin et al., 2019; Karyotaki et al., 2020; Pascoe et al., 2020). All three types of stress were also associated with lower well-being for both genders, specifically lower self-acceptance, life satisfaction, self-esteem, and social support, findings consistent with previous studies that also found stress to be a threat to well-being (Ribeiro et al., 2018; Barbayannis et al., 2022; Minshew et al., 2023). Stress coping styles were relevant to mental symptoms and well-being for both boys and girls, with a more emotional and less rational stress coping style being associated with more mental symptoms, lower psychological well-being, lower life satisfaction, and lower self-esteem and social support. These findings are congruent with those of previous studies in which problem-focused stress coping styles were found to be healthier than emotion-focused coping styles (Watson and Sinha, 2008; Matud, 2016a; Ben-Zur, 2009).

For both boys and girls, the main predictor of more mental symptoms was a greater emotional coping style, followed by lower self-esteem, a greater number of stressful life events, and greater chronic stress. In addition, for girls, more mental symptoms were associated with more study dissatisfaction and less social support. Although for both genders the main predictor of greater psychological well-being was greater self-esteem, greater rational coping style was relevant, as was greater study satisfaction. Higher study satisfaction also predicted higher life satisfaction, which was also associated with less chronic stress. Taken together, these findings highlight the importance of stress and coping styles for students’ mental health. Age and educational level were not relevant predictors of mental symptomatology. However, for both genders, younger age and higher educational level predicted greater psychological well-being and life satisfaction.

The current study has several limitations. First, this is a cross-sectional study, so no cause-and-effect assumptions can be made. Second, this is a convenience sample, which limits the generalizability of the findings. Third, all measures were self-reported, which is subject to several biases, including recall problems and social desirability. Fourth, personality measures such as extraversion or neuroticism, which previous studies have shown to be related to well-being (Phuthong, 2023), were not included in this study. Finally, all students resided in Spain, so the results cannot be generalized to other countries with different values regarding adolescents and youth education and the possibility of access to university studies. Future studies should be longitudinal, with probability samples, and conducted with students from different countries and cultures. In addition, multi-method measures, including individual interviews, should be included to provide a deeper and more idiographic understanding of students’ main academic and non-academic stressors.

## 5 Conclusion

Despite these limitations, the results of the present study allow us to conclude that although students are exposed to academic stressors, non-academic stressors are common, especially those related to family relationships and conflicts and romantic relationships. An important differentiating factor in students’ stress and mental health is whether they are university students or non-university students. Although university students have more chronic stress than non-university students, they have less stress due to dissatisfaction with their studies. Stress coping styles are healthier for university students, who have better mental health than non-university students. For both genders, the main risk factor for mental symptoms is a higher emotional coping style, followed by lower self-esteem, a higher number of stressful life events and changes, and higher chronic stress. Self-esteem, social support, coping styles, study dissatisfaction, age, and chronic stress are significant factors for students’ well-being. The findings of this study are relevant to the design of policies, psychological programs, and strategies aimed at improving the mental health and well-being of university and non-university students. Central to such programs is the teaching and promotion of healthy stress management. This includes promoting a more problem-focused and less emotion-focused coping style. In addition, strategies to increase self-esteem and social support should be included in such policies and programs.

## Data Availability

The raw data supporting the conclusions of this article will be made available by the authors, without undue reservation.
